# Enhancing Confidence in Evidence-Based Trauma Care and Implementation Research: Training Program for Clinicians in Ukraine

**DOI:** 10.21203/rs.3.rs-6341493/v1

**Published:** 2025-07-28

**Authors:** Tetiana Nickelsen, Gregory N. Muller, Shauna L. Clark, Oleksandr Bordiuzhenko, Israel Liberzon, Marcia Ory

**Affiliations:** Texas A&M University; The University of Texas at Austin; Texas A&M University; Texas A&M University; Texas A&M University; Texas A&M University

**Keywords:** PTSD, intervention, evidence-based, implementation science, Ukraine, mental health

## Abstract

Since the Russian invasion in February 2022, millions of Ukrainians have been exposed to war-related trauma, with projections indicating hundreds of thousands of individuals will develop debilitating trauma/stressor-related disorders. Ukraine faces a critical shortage of mental health professionals trained in evidence-based care (EBC) for post-traumatic stress disorder (PTSD), and even fewer with expertise in implementation research. This study examined changes in clinicians’ confidence in implementing evidence-based trauma care and conducting implementation research following a comprehensive training program. Forty-one Ukrainian mental health professionals attended a five-day training workshop in Lviv, Ukraine, covering evidence-based PTSD treatments and implementation research frameworks. Participants completed pre- and post-training surveys that assessed their confidence levels and perceived barriers. Data was analyzed using Wilcoxon Signed-rank tests, multiple regression, and thematic analysis. Participants demonstrated significant increases in confidence in both implementing evidence-based trauma care and conducting implementation research. Training efficacy was independent of professional background and years of experience. Thematic analysis identified key barriers to implementing EBC and in conducting implementation research. Findings highlight the need for continued effort to address the identified barriers for adapting EBC in a Ukrainian context. This training model may serve as a foundation for developing a sustainable mental health workforce capable of addressing the severe trauma burden in Ukraine.

## 1. BACKGROUND

Since the Russian invasion in February 2022, millions of Ukrainians have been exposed to combat and war-related traumas (1). It is projected that hundreds of thousands of people in this region are likely to develop debilitating trauma/stress-related mental health sequela (2, 3). At the same time, there is a dearth of mental health professionals in Ukraine who are trained in evidence-based care (EBC) for anxiety, depression, and post-traumatic stress disorder (PTSD) (4). There are even fewer investigators who can implement, disseminate, and assess the fidelity, acceptability, and efficacy of these treatments within the context of resource-limited Ukraine (5, 6). Taking into consideration the substantial number of Ukrainians who already have or are likely to develop PTSD and other trauma-related conditions/disorders in the foreseeable future, there is an acute need for appropriately trained clinician-researchers. The long war exposure underscores not only the need for clinician-researchers, but also an understanding of how the changing context will impact treatment needs. This will require adaptation of state-of-art tools imported from elsewhere, and continuous scientific evaluation to optimize both interventions and implementation practices for the evolving circumstances within Ukraine. It is, therefore, of critical importance to train Ukrainian clinician-researchers in basic stress biology and post-traumatic EBC strategies. Emphasis should also be given to best practices for community-level mental health care needs assessment, implementation science, and program evaluation. Data will be needed to shape treatments for optimal impact in this context and to refine training programs for the most efficient skill development in this (and other) international contexts.

Over the last decades, a number of empirically validated evidence-based treatments for the consequences of trauma have been developed and implemented. Evidence-based PTSD treatment in adults includes specialized therapeutic approaches like prolonged exposure therapy (7), transdiagnostic behavior therapy (8), cognitive processing therapy (9), pharmacotherapy (10), eye movement desensitization and reprocessing (11), and more. These approaches have demonstrated efficacy in addressing the complex symptoms of PTSD and trauma-related conditions, with prolonged exposure often referred to as the “gold standard” for the treatment of PTSD (7). However, while effective treatments have been implemented in many settings, there is still a need to improve evidence-based treatments and their implementation. Only 60 to 70 % of those treated have clinically meaningful improvement (12), and the success rate is even less known in active war zones.

While EBC is critical for providing a standard of care, implementation research is crucial for understanding program outcomes and examining processes, strategies, and contextual factors that influence the successful application of interventions (13). Beyond program effectiveness, implementation research investigates real-world challenges and facilitators affecting the delivery and sustainability of adopted strategies. It illuminates the mechanisms underlying success or failure, helping refine interventions and offers insights for policymakers, practitioners, and researchers. By addressing contextual nuances, it informs the development of strategies to enhance implementation fidelity, ultimately contributing to evidence-informed decision-making and fostering effective, sustainable interventions in diverse settings.

This paper describes a programmatic effort to remediate the lack of trained mental health providers in evidence-based trauma care while simultaneously enhancing the providers’ understanding of how to best implement these interventions within their existing structures. This effort developed a training program for evidence-based trauma-focused treatment and implementation/dissemination research in Ukraine, funded by a training grant from the National Institutes of Health (NIH) Fogarty Center. Our project not only provides foundational training in evidence-based therapeutic interventions, but also incorporates key elements of implementation research, which is often neglected in resource-limited settings. By simultaneously training clinicians in EBC and the science of translating these practices into real-world settings, our goal is to create a sustainable model for improving trauma care delivery in Ukraine. Moreover, the program addresses the long-term need for established clinician-researchers who can continually adapt and refine trauma care strategies as the socio-political landscape in Ukraine evolves. This will support the sustainable and effective implementation of EB in the face of ongoing challenges.

The current manuscript reports initial data on a cohort of Ukrainian learners who underwent this training in 2023. The current study objectives were to examine the change in confidence and familiarity of our trainees from pre- to post- training with respect to (a) implementation of EBC and (b) engagement in implementation research. We also explored whether participant characteristics were related to efficacy perceptions. Additionally, we assessed trainee’s perceptions of the barriers related to these processes.

## 2. METHODS

### 2.1 Participants

The identification of candidate participants for the workshop adhered to specified criteria including (a) being a practicing clinical psychologists or psychiatrists with a familiarity with cognitive behavior therapy, (b) currently treating PTSD patients, (c) demonstrated interest in implementational research, and (d) sufficient command of the English language to ensure effective learning during training. In consideration of the three distinct principal systems of psychological assistance in Ukraine (i.e., governmental clinics, public/private practices, and military mental health system), the number of training slots (limited to ~40 participants) was equitably distributed among these three sectors. Through our in-country Ukrainian partners, we received 350 applications of interest which were further screened in a second phase of online interviews. From the 150 applicants passing the second stage, a total of 41 trainees participated in the study, with 39 respondents completing the questionnaire, yielding a 95% response rate. Of these, 74.36% completed the training in person, and 25.64% attended via the online Zoom platform.

Participants had a variety of professional backgrounds, including psychologists, psychiatrists, and psychiatrists with a minor in psychology. The sample was predominantly female. Geographically, participants were from a broad range of regions across Ukraine, with the largest number coming from Kyiv Oblast, followed by Lviv Oblast, and smaller groups from Kharkiv, Vinnytsia, Chernivtsi, Sumy, and Kropyvnytskyi Oblasts. In terms of professional experience, participants had a wide range of experience, with some having just a few years in the field, others having moderate experience, and a significant portion with over a decade of experience (see Table 1).

The first in-country training session was conducted in Lviv, situated in the Western part of Ukraine. This selection was made primarily based on safety considerations with respect to the frequency of nightly air raids.

### 2.2 Training components.

The comprehensive five-day workshop program encompassed the following thematic modules: (a) Psychological trauma, stress, and PTSD, (b) Introduction to Implementation Research, (c) Introduction to Cognitive Processing Therapy, (d) Training module in Prolonged Exposure Therapy, (e) Training module in Transdiagnostic Behavioral Therapy, (f) Pharmacological therapy for PTSD, (g) Neurobiology of PTSD, (h) PTSD Differential diagnostic assessment, (i) PE in real settings of deployment/combat, and (j) Acute Post-trauma Interventions (See Additional file 1). Two trainers were native Ukrainian speakers and at least one Ukrainian speaking trainer was present at all training sessions.

*Prolonged exposure* (7) involves the systematic and safe re-exposure to trauma-related memories, reminders, and situations that are usually avoided by PTSD patients. This exposure helps individuals to process (and thereby reduce) the emotional distress associated with traumatic experiences.

*Transdiagnostic behavior therapy* (8) takes a broader perspective by targeting common underlying mechanisms across various emotional disorders, including PTSD. This approach addresses maladaptive behaviors and thought patterns, promoting adaptive coping strategies applicable to a range of symptoms.

These therapies backed by extensive research and have demonstrated effectiveness in alleviating symptoms, improving functioning, and promoting long-term recovery. Additionally, their structured and systematic nature allows for a tailored and individualized approach to address the unique needs of each person experiencing PTSD.

#### Pharmacotherapy.

Similarly, pharmacotherapeutic agents with a demonstrated efficacy against PTSD symptoms were reviewed, as an effective alternative if prospective patients are unable or unwilling to engage in psychotherapy.

#### Implementation research framework (14).

The introduction to implementation research training comprised two, 2-hour sessions. The training focused on equipping participants with the skills necessary to conduct implementation research. Ultimately, the goal was to cultivate a group of clinician-researchers who not only understood the theoretical and empirical bases for EBC but were also prepared to identify, analyze, and address the unique barriers to its successful implementation in their specific settings.

Specifically, this was achieved by:

1. Providing a theoretical framework of implementation science (14).
2. Introducing trainees to a variety of implementation frameworks and tools (e.g., PRISM and RE-AIM framework) that guide the identification of barriers at multiple levels, including individual, organizational, and systemic (14).
3. Offering practical case studies and real-world examples that highlighted the kinds of barriers encountered in different contexts, with opportunities for trainees to analyze and discuss these challenges in group settings. By simulating the complexities of EBC implementation, trainees were encouraged to think critically about potential obstacles they may encounter in their own practice (15).
4. Developing problem-solving skills by encouraging trainees to propose solutions to these barriers. This process involved using evidence from existing research on effective strategies for overcoming implementation challenges, such as adapting interventions to local needs, fostering organizational support, and addressing clinician attitudes toward EB practices (16).
5. Providing opportunities for trainees to collaborate and brainstorm solutions in small groups, promoting the development of a collective understanding of the factors that may hinder implementation and the potential ways to mitigate these challenges. Mentorship from experts in the field also played a critical role in guiding trainees through the complexities of finding contextually appropriate solutions (17).
6. Fostering an ongoing process of reflection and adaptation, encouraging trainees to consider how they might reassess and refine their strategies for overcoming barriers in response to evolving circumstances or new information. In doing so, we aimed to build a mindset of continuous evaluation and adaptation, critical for sustained EBC implementation (18).

### 2.3. Assessment

Participants completed pre-assessment questionnaires before the training program’s commencement. In-person participants received paper questionnaires and virtual participants received a survey via Google Forms. The same set survey was then administered as a post-assessment immediately fowling the completion of the training. Participants were given sufficient time to complete the questionnaires independently, ensuring the confidentiality of their responses.

The pre- and post-assessment questionnaires captured participants’ background, profession, years of experience, geographic region, and current engagement in different types of EBC as well as self-reported measure of participants’ familiarity with EBC and implementation research. Perceived confidence was also captured related to participant’s ability to apply evidence-based approaches and engage in implementation research after training. Confidence level was assessed on a 4-point Likert scale of “Not at all”, “Very little” “Somewhat confident” and “To a great extent.” Included questions also inquired about expected barriers to EBC and implementation research.

### 2.4. Analytic Plan

All analyses were conducted using Statistical Package for the Social Sciences (SPSS) programming. Descriptive statistics, including frequencies and percentages, were used to analyze the participants’ demographics and engagement in EBC. Wilcoxon Signed-ranks test was selected to identify changes in confidence in engaging in EBC and implementation science before and after the training. To examine whether trainees’ characteristics affected the results of the training, we selected two trainees’ characteristics that might play an important role in determining the efficacy of our training, as expressed through their confidence in engaging in EBC and implementation science. These variables were chosen as they are potentially modifiable (i.e. determined during selection of potential trainees). The first variable is a discipline in which trainee was trained (psychiatrist vs clinical psychologist). The second variable is the trainees’ level of experience, expressed in terms of how many years they have been in clinical practice. We used multiple regression where years of trainees’ experience, or profession, separately predicted trainee’s confidence after the training, controlling for pre-training confidence levels. We also examined whether years of experience predicted confidence while controlling for the trainee’s profession.

We employed thematic qualitative analysis (19) to identify themes from open-ended responses regarding (a) barriers to EBC and (b) barriers to implementational research. Thematic analysis was conducted following Braun & Clarke’s framework, involving familiarization with the data, generating initial codes, searching for themes, reviewing themes, defining and naming themes, and writing up results. Themes were categorized based on recurring patterns and counted to determine their prevalence. Frequencies of responses for each theme were quantified and visualized to highlight their relative importance. We identified the perceived barriers to providing EBC practices in trauma care in Ukraine through the question: “What do you see as the greatest barriers to implementing evidence-based practice in trauma care in Ukraine?” Trainees were asked to identify one or two barriers. Thirty-three of thirty-four trainees responded to this question. To identify barriers to engaging in implementational research on trauma care, we used the question, “What do you see as the greatest barriers to engaging in implementational research on trauma care in Ukraine? Trainees were asked to identify one or two barriers. Thirty-one of thirty-four trainees responded to this question.

## 3. RESULTS

### 3.1. Pre- and post-workshop confidence in engaging in evidence-based practices on trauma care. (Figure 1a)

Prior to training, a significant portion of participants reported low confidence in engaging in evidence-based trauma care, with many reporting either “not at all” or “very little” confidence. The majority (56.4%) felt “somewhat” confident, while only a small percentage reported being confident “to a great extent.” After the training, no participants indicated low confidence, and the majority (71.8%) reported feeling “very confident,” while a smaller group felt “somewhat confident.” This shift demonstrates a substantial increase in confidence following the training. The Wilcoxon Signed-ranks test revealed a significant pre-post difference in confidence (Z=−4.40, p<.001), highlighting the effectiveness of the training in enhancing participants’ confidence in engaging with evidence-based trauma care.

### 3.2. Pre- and post-workshop confidence in engaging in implementation research on trauma care. (Figure 1b)

We also examined changes in confidence regarding engaging in implementational research on trauma care before and after the training. Prior to the training, the majority of participants felt either “very little” or “somewhat” confident, with only a small group feeling “to a great extent” confident. After the training, confidence levels showed a marked improvement. The proportion of participants reporting “somewhat” confidence increased significantly, while the number of those feeling “not at all” or “very little” confidence dropped substantially. Notably, the number of participants feeling “to a great extent” confidence rose significantly (from n=5 to n=23), reflecting a strong increase in confidence in engaging with evidence-based research on trauma care. The Wilcoxon Signed-ranks test revealed a significant pre-post difference (Z=−3.74, p<.001), demonstrating the effectiveness of the training in boosting confidence in this area.

### 3.3. Trainees’ Characteristics and Confidence Levels.

To examine whether selected characteristics predicted post- training confidence level we used multiple regression analyses to examine the associations between trainee characteristics and confidence perceptions. Years of experience did not predict post-training confidence levels, controlling for pre-training levels for either EBC (b=0.073, p=0.59) or implementation research (b=−0.025 p=0.89). Similarly, type of professional training did not predict confidence in engaging in EBC (b=0.023 p=0.91) or implementation research (b=0.219, p=0.56). Years of experience did not predict post-training confidence (b=0.074, p=0.60), even after controlling for professional training background.

### 3.4. Perceived Barriers. (Figure 2a and 2b)

**Figure 2:**
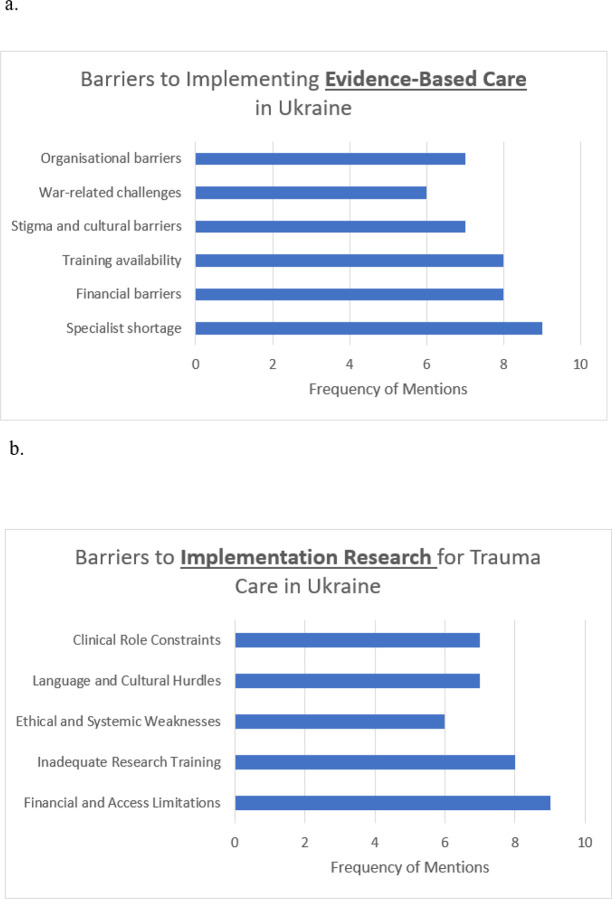
Barriers to implementing EBC (2a) and implementation research (2b) for trauma care in Ukraine.

Thematic analysis of the perceived barriers to providing EB practices in trauma care identified five key themes: Specialist shortage (n=9), Training availability (n=8), Stigma and cultural barriers (n=8), War-related challenges (n=7), Financial barriers (n=6). Additionally, five responses were categorized as “Other,” encompassing barriers not fitting neatly into predefined categories. (Figure 2a).

1. Specialist shortage. This theme was the most frequently mentioned and identified lack of specialists trained in evidence-based methods. E.g., “*With too few providers for too many patients, we struggle to keep up with the evidence*-*based guidelines while managing immediate clinical demands*.”
2. Training availability. Included barriers such as insufficient training programs and inadequate access to educational opportunities. Participants expressed concerns about the scarcity of evidence-based training: “*Not enough clinics provide training, and the education available for psychiatrists is often too expensive*.”
3. Systemic and Organizational Barriers. Seven responses highlighted systemic and organizational challenges, including insufficient regulation, lack of financial resources, and limited institutional support. One participant noted: “*There is no state regulation of psychotherapeutic practice, which makes it difficult to standardize care*.”
4. Stigma and cultural barriers. Stigma emerged as a significant barrier. Participants identified negative perceptions of mental health care and a lack of public awareness about trauma and PTSD. For instance: “*People don’t know that their complaints are symptoms of PTSD, and stigma around mental health prevents them from seeking help*.
5. War-related challenges. Seven responses cited the ongoing war and associated socio-economic challenges as critical barriers. The continued conflict in Ukraine has created unique pressures that hinder the implementation of EBC: “*The ongoing war makes it difficult to focus on long*-*term studies to become evidence*-*based practitioners*”
6. Financial barriers. Although mentioned less frequently, financial barriers for patients were highlighted as a significant concern. One participant stated: “*In Ukraine, access to EBC is often limited to expensive private clinics, making it unaffordable for many patients who rely on an overburdened public healthcare system*.”

The results underscore the multifaceted challenges to implementing evidence-based trauma care in Ukraine. Key barriers such as training deficiencies, systemic hurdles, and cultural stigma must be addressed to improve mental health care outcomes.

Using thematic analysis of perceived barriers for engaging in implementational research in trauma care in Ukraine, we identified five key themes: Financial and Access Limitations (n=9), Inadequate Research Training (n=8), Ethical and Systemic Weaknesses (n=6), Language and Cultural Hurdles (n=7), and Clinical Role Constraints (n=7). More form information, see Figure 2b. Additionally, seven responses were categorized as “Other,” encompassing barriers not fitting neatly into predefined categories.

1. Financial and Access Limitations. A primary barrier identified was the lack of funding and restricted access to academic resources. Participants highlighted that securing financial support for research is extremely difficult, with minimal investment from government or private institutions. One respondent stated: “*There are simply no funds allocated for medical research. Scientists are underpaid, and grants are virtually nonexistent*.” Additionally, access to international journals and research databases is limited due to high subscription costs. This financial barrier hinders the ability to stay updated with global advancements in trauma care.
2. Inadequate Research Training. Another significant theme was the insufficient education and mentorship in research methodologies. Universities primarily focus on clinical skills, with little to no emphasis on research training. One of our trainees shared: “*We are taught to be doctors, not researchers. Research skills are a very small part of our curriculum*.” Furthermore, the absence of experienced mentors to guide young researchers was cited as a key issue, leading to a lack of proper training in research design, data analysis, and scientific writing.
3. Ethical and Systemic Weaknesses. Systemic barriers, including weak medical research standards and lack of ethical oversight, were also prominent. Participants noted the absence of structured ethical guidelines, contributing to poor research integrity and quality. One clinician observed: “*There is no standardized ethical review process. Many studies are conducted without proper approvals*.” Additionally, respondents expressed concerns about corruption, further weakening the research environment.
4. Language and Cultural Hurdles. Language and cultural factors significantly impact research participation. Many Ukrainian researchers lack proficiency in English, making it difficult to publish in high-impact international journals. One trainee explained: “*Even if I conduct a good study, my lack of English proficiency makes it almost impossible to publish in respected journals*.” Additionally, there is a resistance to EBC among some clinicians, who prefer traditional methods over scientific advancements. This cultural attitude discourages research initiatives and hinders the adoption of new findings into clinical practice.
5. Clinical Role Constraints. Finally, structural and professional expectations limit clinicians’ involvement in research. Many hospitals and institutions prioritize patient care over research, making it difficult for clinicians to allocate time for scientific studies. One clinician stated: “*We are expected to treat patients, not conduct research. Research is seen as a luxury, not a necessity*.” Employers are often reluctant to allow clinicians to dedicate work hours to research activities, further restricting opportunities for scientific contributions.

## 4. Discussion

Research consistently demonstrates that structured training can improve clinicians’ knowledge, attitudes, and confidence regarding evidence-based trauma care (20, 21). Similar to our findings, Karlin et al.(22) reported significant improvements in self-reported confidence and readiness to deliver evidence-based psychotherapies following intensive training programs for Veterans Affairs clinicians. While various PTSD treatment training workshops have been introduced in Ukraine since the invasion in 2022, their effects on increasing provider knowledge, skill acquisition, competence, adherence, or satisfaction remain largely unknown, creating a crucial gap in our understanding of training effectiveness in conflict settings. Our study addresses this gap by assessing the impact of a recently developed week-long training program on participants’ confidence in engaging with evidence-based trauma care and implementation research.

Our findings demonstrate that confidence in engaging in EBC for PTSD and in implementational research significantly increased from pre- to post-training. This aligns with previous research by Ruzek et al. (23), who found that structured trauma training improved clinicians’ self-efficacy in delivering evidence-based PTSD treatments. While confidence in EBC before training varied among our trainees, there was overall significant and meaningful improvement in confidence across all, or almost all, participants (see Results). This finding is of particular importance because, as suggested by Aarons et al. (24), provider attitudes and confidence are crucial factors in determining the successful adoption of EBC. We believe that at least some confidence in EBC is required in order to engage in this practice; follow-up analysis of second-year data will be able to test this important assumption, as supported by implementation science frameworks (16, 25).

Our work offers preliminary evidence that the training we developed for Ukrainian practitioners might be useful despite multiple challenges stemming from the ongoing active war, the cultural/language barriers, the compressed training schedule and more. While other types of trainings for trauma care, with varying degrees of empirical support, are being currently conducted across Ukraine, this is the first empirical evidence for the effectiveness of training in EBC in this context. Similar challenges have been documented in other conflict-affected regions, where Murray et al. (26) found that adapting evidence-based trauma treatments to local contexts while maintaining fidelity was both possible and effective. Naturally, future studies examining direct engagement in EBC are needed to confirm these preliminary findings, a recommendation consistent with the stepped implementation approach advocated by Powell et al. (27).

Similar to confidence in EBC, the level of participant confidence in engaging in implementation research significantly improved following the training. This is an important point because training in implementation and dissemination science is not an integral part of training for Ukrainian clinicians/scientists. Moreover, overcoming multiple cultural, language and other barriers to introduction of EBC in Ukrainian environment requires good understanding of the principles and the methods of implementation science. Lyon et al. (28) similarly found that implementation science training was essential for clinicians working in low-resource or complex service environments and Baumann et al. (29) demonstrated that implementation science training improved researchers’ and practitioners’ ability to address implementation barriers. Improving confidence in implementation science is particularly important if adoption of EBC in Ukraine is a long-term goal. While the majority of trainees are not expected to become implementation researchers, a primary aim is building teams led by individuals with strong backgrounds in implementation science to study the use of EBC in Ukraine, following models described by Moullin et al. (30).

This study also examined the effects of individual characteristics of our trainees on the effectiveness of the training offered. Two key variables could have affected the efficacy of training, and that may have potentially adjusted the initial selection of the trainees entering the training. Our preliminary findings suggest that the training effects are independent of professional background (Psychiatrists vs Clinical Psychologist) and did not vary with number of years practicing. This aligns with findings from Beidas & Kendall (31), who found that clinician demographic characteristics were less predictive of training outcomes than organizational factors and implementation climate. We note that due to a relatively small number of trainees this analysis may have been underpowered, potentially leading to type 2 error, a limitation also noted by Stirman et al. (32) in similar implementation studies. As such, the results are considered preliminary. Findings are noteworthy as they suggest that there is no need to adjust selection criteria for training enrollment. Until additional evidence is available, these preliminary findings suggest that the training effects are not discipline-specific and that even experienced therapists are interested and willing to adopt effective novel treatments in Ukraine.

We collected information about perceived barriers for implementing EB trauma care and engaging in implementational research in Ukraine. The lack of qualified specialists is the most frequently cited barrier for both providing EB trauma care and engaging in implementation research. This mirrors findings by Romaniuk (33), who identified workforce capacity as a critical barrier to mental health care in Ukraine prior to the current conflict. A strong support for this program to train more specialists and to collaborate with Ukrainian universities to help them develop similar training programs within their curriculum. The next cluster of barriers listed by our trainees is the financial challenges, stigma and institutional barriers. Similar barriers have been documented by Semrau et al. (34) in their examination of global mental health capacity building initiatives in low- and middle-income countries. These barriers are geographically specific and thus might require adapting the EBC to the realities on ground in particular geographic regions of Ukraine. The appropriate adaptation of EBC requires good knowledge and familiarity with implementation and dissemination efforts, as emphasized by Aarons et al. (35) in their discussion of the dynamic adaptation process. This further underlines the importance of implementation science in EBC training, consistent with recommendations by Chambers & Norton (36) for context-sensitive implementation approaches.

### 4.1 Limitations.

A primary study limitation is the relatively small number of trainees involved. This limits the ability to generalize study findings, especially when the analyses involve sub-groups (e.g., physicians vs. psychologists). Further, our findings are limited to potential trainees with a relatively fluent command of the English language. Many clinicians in Ukraine are less than fluent in English and will likely have additional challenges when assimilating novel evidence-based approaches. Our findings also demonstrated changes in our trainee’s confidence immediately after the training, and it is not known how long these effects will persist post-workshop. Finally, collected data reflects our trainees’ subjective reflections and their own assessment of their confidence. While important, these findings do not speak about the actual translation of our trainee’s confidence into changes in their treatment approaches. Longitudinal prospective studies will explore the participants’ use of EBC to help to address this important question.

## 5. Conclusion

This training series appears initially efficacious in increasing the confidence of our trainees in engaging EBC and implementation research. The efficacy of training appears to be independent of the trainee’s background. We identified key perceived barriers for implementing EBC and Implementation science as seen by our trainees.

## Supplementary Files

This is a list of supplementary files associated with this preprint. Click to download.

- Additionalfile1.docx

## Figures and Tables

**Figure 1:**
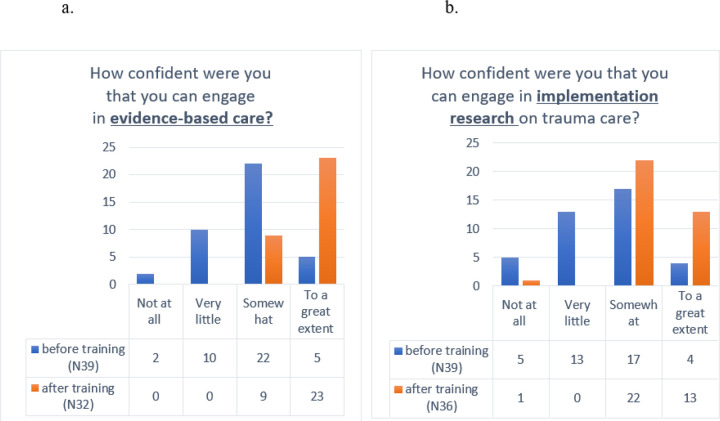
Pre- and post-workshop confidence in engaging in EBC (1a) and in implementation research (1b).

**Table 1.** Participants characteristics.

Demographic of Interest		N (%)
Format	In-person	29 (74.36%)
	Online	10 (25.64%)
Sex	Female	26 (66.67%)
	Male	13 (33.33%)
Profession	Psychology	18 (46.15%)
	Psychiatry	6 (15.38%)
	Psychiatry with additional training in Psychology	15 (38.46%)
*Background	Clinical	39 (100%)
	Academic	22 (56.41%)
	Military	4 (10.26%)
	Administrative	4 (10.26%)
Years of experience	1–2 years	7 (17.95%)
	3–5 years	8 (20.51%)
	6–10 years	12 (30.77%)
	10+	12 (30.77%)
Geographic region	Kyiv	15 (38.6%)
	Lviv	8 (20.51%)
	Kharkiv	3 (7.69%)
	Vinnytsia	2 (5.13%)
	Chernivtsi	2 (5.13%)
	Kropyvnytskyi	2 (5.13%)
	Sumy	2 (5.13%)
	Poltava	2 (5.13%)
	Ternopil	2 (5.13%)
	Ivano-Frankivsk	2 (5.13%)
	Khmelnytskyi	2 (5.13%)
	Kherson	2 (5.13%)

Note: n=41 participated in the training, 39 filled in questionnaires

* Overlap within groups

## Data Availability

The datasets generated and/or analyzed during the current study are available from the corresponding author upon reasonable request.

## List of abbreviations

EBC: Evidence-Based Care
PTSD: Post Traumatic Stress Disorder
PE: Prolonged Exposure
CPT: Cognitive Processing Therapy
TBT: Transdiagnostic Behavior Therapy
